# A Case of Cardiac Calcified Amorphous Tumor Presenting with Concomitant ST-Elevation Myocardial Infarction and Occipital Stroke and a Brief Review of the Literature

**DOI:** 10.1155/2017/8578031

**Published:** 2017-12-24

**Authors:** Kyaw Kyaw, Htun Latt, Sammy San Myint Aung, Chanwit Roongsritong

**Affiliations:** ^1^Institute for Heart and Vascular Health, Renown Regional Medical Center, 1500 E. 2nd St. No. 302, Reno, NV 89502, USA; ^2^Department of Internal Medicine, University of Nevada-Reno, School of Medicine, 1155 Mill St. No. W11, Reno, NV 89502, USA

## Abstract

Cardiac calcified amorphous tumor (CAT) is an extremely rare benign intracavitary tumor of the heart. It may mimic other cardiac tumors and can present with signs or symptoms of systemic embolization. There are limited data regarding CAT in the literature. We report a case of a 68-year-old woman with a cardiac CAT and mitral annular calcification (MAC), who presented with acute ST-elevation myocardial infarction (STEMI) and occipital stroke. After extensive review of the literature, we believe that this case is possibly the first description of a cardiac CAT presenting with STEMI. The CAT was surgically removed, and the diagnosis was confirmed by histology. The patient tolerated the surgery and reported no events at 6-month follow-up.

## 1. Introduction

Cardiac calcified amorphous tumor (CAT) is a rare benign intracavitary tumor of the heart. Like other cardiac tumors, CAT may present with dyspnea, syncope, and pulmonary or systemic embolization and obstruction [1–4]. Herein, we are reporting a case of CAT with mitral annular calcification, who presented with acute ST-elevation myocardial infarction (STEMI) and occipital stroke. To the best of our knowledge, our case perhaps is the first description of a cardiac CAT presenting with STEMI.

## 2. Case Presentation

**Figure 1:**
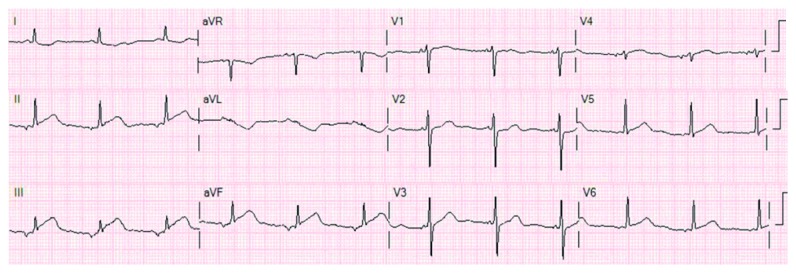
EKG showing ST elevation at II, III, and aVF with reciprocal ST depression at I and aVL.

**Figure 2:**
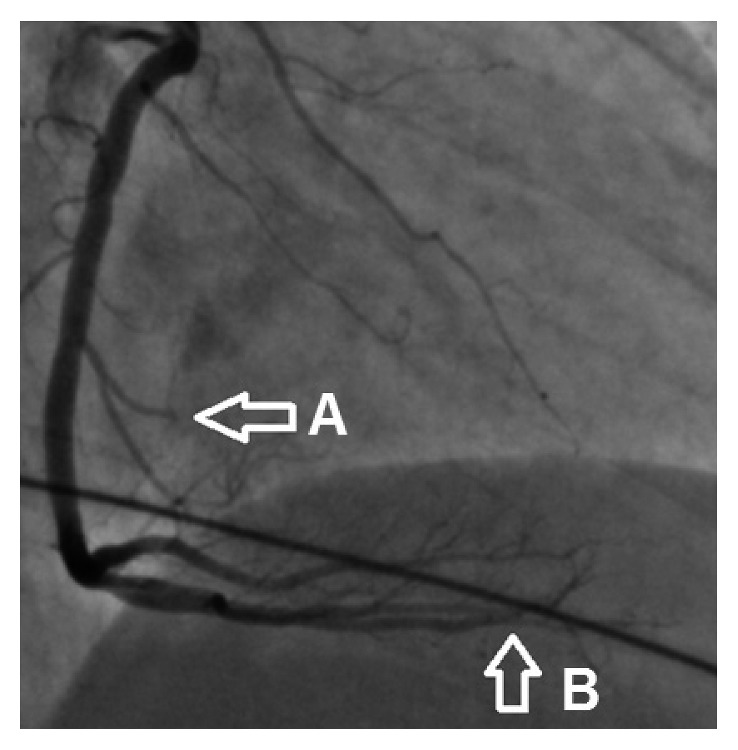
Coronary angiogram showing an occlusion of the small acute marginal branch (A) of the right coronary artery with slow flow in the posterior descending artery (B).

**Figure 3:**
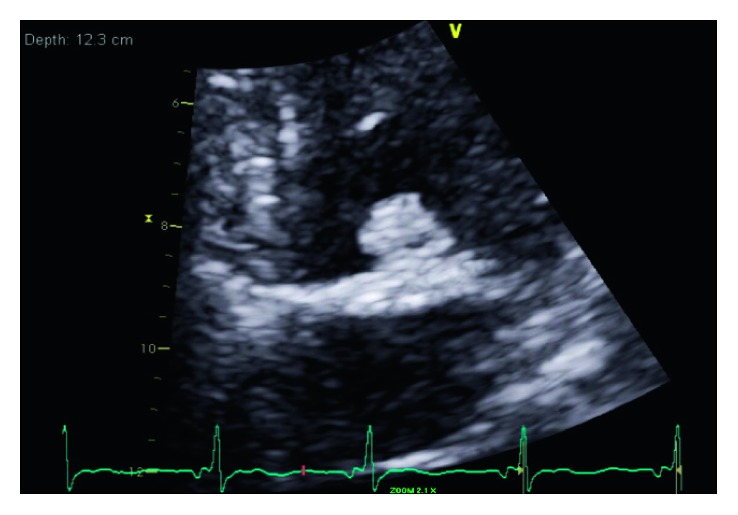
Transthoracic echocardiogram (TTE) showing a 1.2 × 1.2 cm mobile mass attached to the ventricular aspect of mitral valve and hyperechogenic mitral annulus.

**Figure 4:**
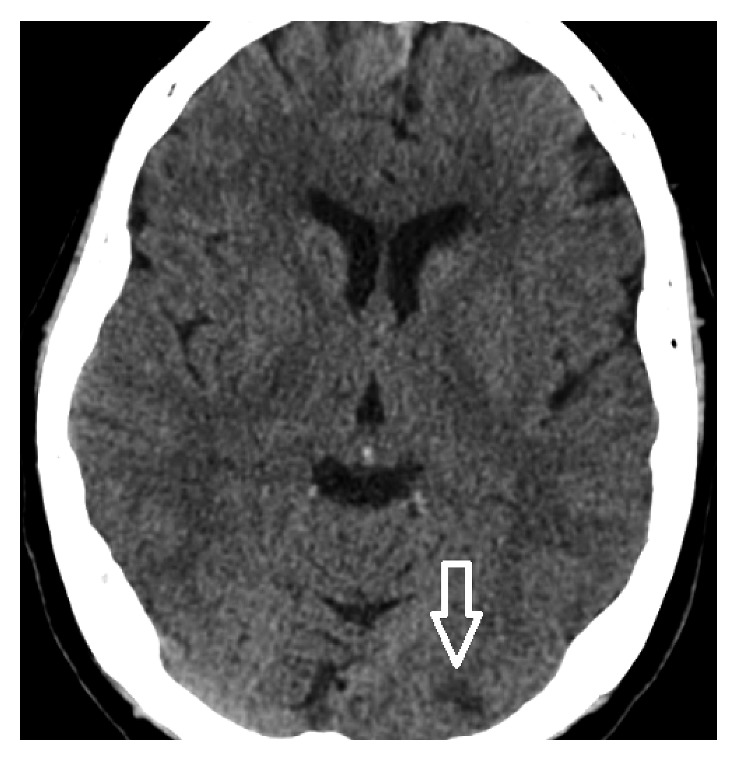
CT scan of the brain without contrast showing an infarct in the left occipital lobe.

**Figure 5:**
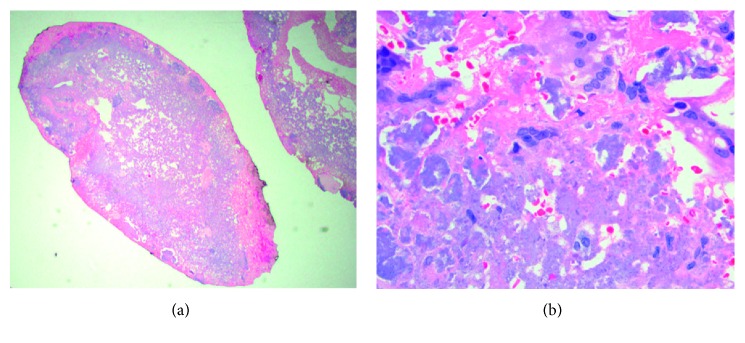
Pathology specimens showing a nodule of amorphous debris and fibrinous material in low-power field (a) and histiocytes and multinucleated giant cells in high-power field (b).

A 68-year-old woman with a 40-pack-year smoking history but no known medical problem was brought in by ambulance for sudden onset of persistent, severe left-sided chest pain without radiation. The patient also reported a new onset of blurred vision. She denied history of hypertension, dyslipidemia, diabetes mellitus, coronary artery disease (CAD), or chronic kidney disease or family history of premature CAD. Initial vital signs were normal with oxygen saturation of 98% on 2-liter supplemental oxygen via nasal cannula. Physical examination was benign, without murmur or abnormal heart sounds. There were no focal neurological deficits or signs of peripheral embolization noted. Initial electrocardiogram (EKG) showed 1-2 mm ST elevation in inferior leads (II, III, and aVF) with reciprocal ST depression in leads I and aVL (Figure 1). Oral aspirin and sublingual nitroglycerin were given immediately. Intravenous heparin was also initiated. Emergent coronary angiography showed an occlusion in one of the acute marginal branches of the right coronary artery and slow flow in the posterior descending artery. There was no other significant CAD (Figure 2). Left ventricular wall motion and systolic function were normal. Subsequent transthoracic echocardiogram (TTE) showed a 1.2 × 1.2 cm mobile mass attached to the base of the posterior mitral valve leaflet on ventricular aspect (Figure 3). Of note, the mitral annulus also appeared rather echogenic. No thrombus was noted in left atrium or left ventricle during TTE or transesophageal echocardiogram (TEE). Troponin I was 1.7 ng/ml on arrival. It increased to 26 ng/ml 10 hours later. Her workups, including complete blood count, prothrombin time/international normalized ratio, calcium, creatinine, liver function test, lipid panel, and hemoglobin A1c, were all normal. CT scan of the brain without contrast showed an infarct in the left occipital lobe (Figure 4). Cardiothoracic surgery was performed. A 1.2 × 0.9 × 0.2 cm tan-pink-colored soft tissue mass was surgically removed without any complications. Histologic examination of the mass showed a nodule of amorphous debris and fibrinous material with histiocytes and multinucleated giant cells without organism, consistent with a cardiac CAT (Figure 5). Throughout the hospital stay, she was closely monitored in coronary care unit and subsequently on telemetry floor. No atrial arrhythmia was reported.

The patient recovered well without recurrent symptoms at her 6-month follow-up.

## 3. Discussion

First introduced by Reynolds et al. in 1997, cardiac CAT is an extremely rare nonneoplastic primary tumor of the heart. It is characterized histologically by the features of calcified nodules and amorphous fibrinous materials [1]. CAT is usually discovered by cardiac imaging studies, but the definite diagnosis requires histological study of the tumor. ElBardissi et al. reported that the lifetime incidence of primary cardiac tumors (benign or malignant) was 0.02% based on 48 years of data collection, and only 8 of 323 cases (2.47%) of primary cardiac tumors were CAT [5]. There have so far been limited data on pathophysiology, treatment, and prognosis of CAT. The majority of CAT is seen in association with valvular heart disease (31%), end-stage renal disease (21%), mitral annular calcification (MAC) (14%), or diabetes mellitus (14%) [2]. Of interest, the MAC-related CAT tends to be highly mobile and more likely to embolize [6]. To date, there have been 13 reported cases of MAC-related CAT [6–11]. Most of them (9 cases) are associated with ESRD. MAC, a chronic fibrous degeneration of the mitral valve, is reportedly caused by abnormalities of calcium-phosphorus (Ca-PO_4_) metabolism [7–9, 12]. MAC has been reported to increase the incidence and mortality of cardiovascular diseases but has not been linked to increased risk of myocardial infarction [13]. MAC is usually diagnosed based on hyperechoic mitral annulus on TTE. Our patient has normal renal function and serum calcium level. The echo densities of the tumor and mitral annulus in our patient are quite similar, suggesting the presence of MAC-like appearance on TTE. We felt that the MAC-like appearance on TTE in our patient and other reported cases without associated ESRD may have resulted from tumor infiltration into mitral valve annulus rather than from the conventional MAC process due to abnormal Ca-PO_4_ metabolism. However, more studies are required to determine the exact underlying mechanism. Unfortunately, as in most patients who underwent surgical resection of the CAT, only the tumor was removed and biopsy of the mitral annulus was not performed in our patient. Surgical resection of the tumor is generally recommended to prevent embolization in most patients [6, 8–10, 14]. Our patient presented with concomitant blurred vision and STEMI, most likely due to the embolic phenomenon from the cardiac CAT.

The patient did not have any evidence of atrial arrhythmias on telemetry monitoring or intracardiac thrombus in TTE or TEE. Additionally, coronary angiogram showed isolated lesions at the third acute marginal branch and PDA of RCA and otherwise nonobstructive coronaries. Carotid ultrasound also showed normal bilateral vertebral and subclavian arteries, but there were mild stenoses (<50%) of bilateral internal carotid arteries (ICAs). Mild ICA stenoses could not explain the left occipital stroke as well. The occluded acute marginal branch of the right coronary artery was <1.5 mm in diameter angiographically. Therefore, no coronary intervention or aspiration was performed. The prognosis is generally good after complete removal of the mass lesion [15, 16]. The only recurrence was reported in a patient who received incomplete resection of the tumor [17]. Our patient tolerated the surgical procedure and has done well without any recurrent events at the six-month follow-up.

## 4. Conclusion

CAT of the heart is rare. Despite its benign nature, it can be highly mobile and consequently lead to systemic embolization, particularly in those with associated MAC. The prognosis is excellent with surgical resection in most patients. For those without ESRD or abnormal calcium metabolism, further studies may help determine whether cardiac CAT can involve the mitral annulus and result in an appearance similar to MAC on TTE.
